# The Feasibility of Elimination of Monkeypox Virus in Nigeria: A Systematic Review

**DOI:** 10.7759/cureus.61867

**Published:** 2024-06-07

**Authors:** Adewale Lawrence, Joseph Anejo-Okopi, Babatunde Adeseye

**Affiliations:** 1 Pharmaceutical Medicine, Bioluminux Clinical Research, Naperville, USA; 2 Microbiology, Federal University of Health Sciences Otukpo, Otukpo, NGA; 3 Clinical Research, Bioluminux Clinical Research, Naperville, USA

**Keywords:** spillover, epidemiology, mpxv, elimination, monkeypox virus

## Abstract

Monkeypox is a zoonotic virus with an increasing incidence in Nigeria, posing significant public health challenges. The virus, related to smallpox, primarily spreads through direct contact with infected animals or humans and has been noted for its potential for wider transmission due to changing ecological and social dynamics. This research aims to evaluate the feasibility of eliminating monkeypox in Nigeria through integrated approaches involving vaccination, public health strategies, and ecological management. Additionally, it seeks to propose a unified public health strategy, incorporating One Health principles, to achieve the elimination of monkeypox in Nigeria. A review of the feasibility of eliminating the monkeypox virus in Nigeria was conducted using databases like Science Direct, Cochrane, PubMed, and Medline. The search was guided by the methodologies and reporting guidelines of the Preferred Reporting Items for Systematic reviews and Meta-Analyses (PRISMA). The initial search yielded 89 publications, but the number of articles that required examination was reduced after applying selected keywords and evaluating abstracts. We deemed 32 articles relevant to the subject after applying inclusion and exclusion criteria. The findings are analyzed, highlighting their limitations and strengths and discussing practical implications, knowledge gaps, and recommendations for future research. This study provides evidence supporting the feasibility of the elimination of the monkeypox virus in Nigeria. Strategic recommendations for a sustainable public health approach to eliminating monkeypox emphasize preventive measures, community engagement, and ecological preservation. This study will provide critical insights into the feasibility of eliminating monkeypox in Nigeria, offering a model that can be adapted for other regions facing similar challenges. The integration of health, ecological, and community-focused strategies is expected to contribute significantly to global efforts to control and potentially eradicate monkeypox. This research could serve as a foundational study for public health authorities in Nigeria and internationally, informing policy and operational decisions to control and eliminate monkeypox as a public health threat. The monkeypox virus reemerges in Nigeria, increasing mortality rates. Awareness programs should be conducted on its danger, transmission mode, and potential human transmission. Public education on the lack of treatment and vaccines is crucial. Meat inspection laws should be enforced to ensure safe animal consumption.

## Introduction and background

Monkeypox, a viral zoonotic disease, first appeared in Nigeria in 1971 and is one of numerous emerging and reemerging infectious diseases globally [1]. Monkeypox is classified under the same genus as the virus responsible for smallpox. Various viruses fall under this category, such as cowpox, horsepox, camelpox, and alaskapox. The variola virus is widely recognized as the most prevalent and extensively studied member of this genus of viruses [2]. Monkeypox, originating from Africa, has become an inclusive alarm, with cases reported in the US, the UK, Israel, and Singapore, affecting Western and Central African clades [3]. Zoonotic, nosocomial, and sexual transmission are all potential vectors for the spread of monkeypox, which can also happen through animal-human or human-to-human contact through respiratory droplets, bodily fluids, and exudates from skin lesions [4]. Lymphadenopathy and systemic rash are the hallmarks of monkeypox, a disease with a low mortality incidence (between 0% and 11%), especially among unvaccinated children and teenagers. One potential danger factor is eating bush meats [3]. While most cases of monkeypox virus infection in humans resolve on their own, monkeys may also be susceptible to latent infection. Since the transmission of monkeypox has shifted from animals to humans in the 1980s and 1970s to humans to humans in more recent years, the virus has grown more infectious in endemic places, with over 70% of human cases occurring in this manner [2]. Due to transmission restrictions, illness symptoms, and a lack of data on putative reservoir species, monkeypox biosafety monitoring networks in many countries are weak [5]. Monkeypox has evolved in a way that is quite dangerous to humans; thus, there has to be a lot more attention and monitoring of this disease. Improving animal supervision, minimizing imported animals, identifying the monkeypox virus at customs, and boosting monitoring are all crucial behaviors [6]. Monkeypox infection has no clinically validated therapy, but preventative measures can help avoid epidemics. Isolation, covering lesions, and wearing masks are recommended. In extreme cases, medicines like brincidofovir, tecovirimat, and vaccinia immunoglobulin may be used. For high-risk contacts, the Ankara vaccination is suggested. It is safe and helps those with early or compromised immune systems produce more antibodies. To determine the pros and cons of preventative monkeypox vaccination in endemic locations, further research and feasibility studies are required [7].

Enhanced infection control procedures, proper hygiene habits, and vaccination with the vaccinia virus can help control the monkeypox virus. Preventive measures include avoiding infected animals, avoiding interactions with infected animals and humans, using personal protective equipment, isolating sick individuals, and ensuring proper hand sanitation. Smallpox vaccinations, such as ACAM2000, can also be used to control transmission during epidemics [8]. More animal-human contacts and increased international travel have contributed to the current upturn of viral illnesses like monkeypox. Staying away from sick animals, materials, and people is one way to prevent the spread of illness. The resurgence of monkeypox might be caused by a combination of factors, including a lack of progress on specialized vaccinations and an increasing global population, while smallpox immunization has the potential to mitigate this [9].

A more stringent worldwide system of case detection and monitoring is necessary to address the monkeypox epidemic, which is a major public health risk. Nevertheless, the factors propelling the disease’s spread in Nigeria, Africa’s most populous country, remain mostly unknown. Because of this lack of data, lawmakers are unable to create efficient control and preventative measures. Urgent public health control measures are required to contain the virus, limit its transmission, and manage the current worldwide outbreak of monkeypox. To help global health authorities avoid the public health crisis that might result from ignoring this illness, this article lays out the risks of doing so and offers solutions, such as improving disease monitoring. This study assesses the feasibility of eliminating the monkeypox virus in Nigeria by investigating the key epidemiological, socioeconomic, and public health factors influencing the transmission, control, and potential eradication of monkeypox.

## Review

Review methods

We performed extensive and methodical looks at studies that have been published, meeting papers, and lab work that has looked at how well the feasibility of elimination of the monkeypox virus works in Nigeria, adhering to the methodologies and reporting guidelines specified by the Preferred Reporting Items for Systematic reviews and Meta-Analyses (PRISMA) guidelines. This inquiry used the following databases: Science Direct, Cochrane, PubMed, and Medline.

Research Question

Based on the research gap described, here are some potential research questions: “What are the key epidemiological, socioeconomic, and public health factors influencing the feasibility of eliminating the monkeypox virus in Nigeria, and what strategies can be developed to address these factors?”

Development of search strategy

To develop different search combinations, Boolean operations such as “OR” and “AND” were used using the keywords mentioned. A detailed search strategy was employed in which appropriate keywords and database-specific key/indexing terms were used related to (monkeypox OR “monkeypox virus” OR “monkeypox disease”) AND (elimination OR eradication OR control OR prevention). PICO criteria were used to formulate the research question in order to specify the population problem, intervention, comparison (if any), and outcomes that were evaluated and considered in the analysis and synthesis of evidence. PICO criteria were followed.

Population and Problem

The population of interest is individuals residing in Nigeria, particularly those at risk of contracting monkeypox, including both human and animal populations.

Intervention

The intervention of interest is the implementation of comprehensive control and elimination measures aimed at reducing the transmission and ultimately eradicating the monkeypox virus in Nigeria.

Comparison

The comparison could involve assessing the current state of monkeypox control measures in Nigeria compared to established international standards or benchmarks for disease elimination.

Outcomes

The study aims to reduce monkeypox cases in Nigeria, improve surveillance systems, increase vaccination rates, enhance public health infrastructure, and evaluate the economic feasibility of elimination efforts.

During the screening process, we made sure to not include anything that was either plagiarized or written in a language other than English. Titles, abstracts, research styles, and accessibility of full texts were considered when evaluating the publications for the inquiry.

Search strategy

We searched five trial registries and three global and regional conferences for ongoing studies. The abstracts and title pages of publications were screened to assess their relevance to the review topics as well as to determine whether they met the requirements for inclusion or exclusion. Relevant studies were obtained by thoroughly examining the complete text and evaluating its appropriateness for inclusion. Controlled vocabulary and keywords were utilized, along with Boolean operators, to construct search queries. The findings are reported, and implications for healthcare practice, policy, and future research directions are discussed.

Inclusion and exclusion criteria

The study on the long-term health outcomes related to the elimination of the monkeypox virus in Nigeria is included in this study. Randomized controlled trials, observational studies, meta-analyses, real-world evidence studies, studies involving human participants of any age with the outcome of interest, studies published in English or with available translations, and studies published from the years 2014 to 2024 were included in this study. Studies that do not assess the elimination of the monkeypox virus in Nigeria, non-peer-reviewed publications, editorials, commentaries, letters, conference abstracts, and animal studies were excluded. Studies focusing solely on animal models or non-human subjects, studies not available in English or lacking accessible translations, and narrative reviews, letters, editorials, and scientific reports were also excluded.

Outcome measures

The study ranked key outcomes to assess the feasibility of eliminating the monkeypox virus in Nigeria. A standardized data extraction form was used to collect relevant information from selected studies, focusing on their design, year, objectives, and conclusions. Initially, title and abstract screening helped identify studies that met the inclusion criteria, which was followed by full-text screening to select the most relevant papers. Additionally, the report includes data from a government-funded cost-effectiveness study. The implementation of preventive measures such as public awareness campaigns and quarantine strategies is highlighted as crucial. These measures encompass antiviral therapies, vaccinations, public health education, promotion of personal hygiene, and prevention of isolation.

Quality assessment

**Figure 1:**
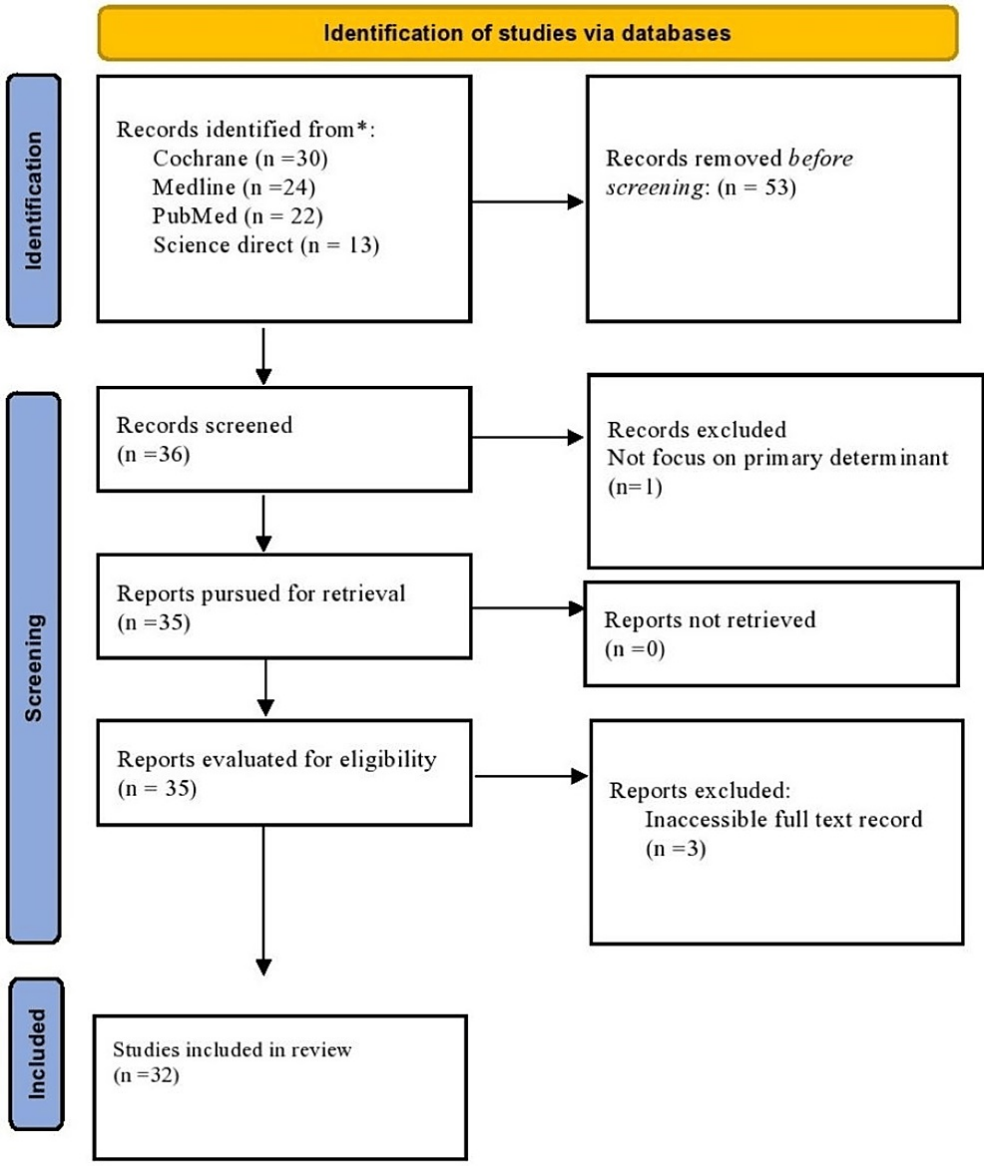
PRISMA flowchart depicting data inclusion strategy for the systematic review PRISMA, Preferred Reporting Items for Systematic reviews and Meta-Analyses

We used the PRISMA system [10] to evaluate research for rigor, study design, and bias risk objectively and comprehensively to guarantee comprehensive, transparent, and fair reporting. Figure 1 explains the PRISMA flowchart for the search and studies selection criteria in detail. Methodological quality and possible bias were assessed using the Newcastle-Ottawa Scale (NOS).

Data synthesis and analysis

Analysis and Synthesis of Data

The synthesis of the extracted data was done narratively, and results regarding the elimination of the monkeypox virus in Nigeria were organized systematically. Subgroup analyses and sensitivity analyses were performed where applicable.

Interpretation of Results

The results were interpreted based on the strengths and restrictions of the studies included in the systematic review. Practical implications and research gaps in the knowledge are explained, along with potential suggestions for future research.

Report Writing

The systematic review manuscript was developed using the guidelines in Figure 1. The paper was organized under proper headings such as Introduction, Methodology, Results, Discussion, and Conclusions.

Critical Appraisal of Studies

The NOS has become a popular tool for assessing study quality due to its simplicity and ease of use. The two included reviews were published between 2000 and 2024. Most studies were of high quality, while others were of medium quality. The NOS assesses the quality of studies based on three main components: selection received 4 points, comparison received 2 points, and outcome received 3 points. The selection component assesses the representativeness of exposed and non-exposed cohorts, ascertaining exposure and demonstrating that the outcome of interest was not present at the start of the study. Comparability evaluates the comparability of cohorts based on design or analysis, considering factors like confounding variables. Outcome assessment evaluates the assessment of the outcome of interest and the adequacy of follow-up duration, considering the ascertainment of outcomes and non-response rates. Each component is scored using predefined criteria, and studies are awarded stars based on their fulfillment, with higher scores indicating higher methodological quality.

Findings of the review

The search yielded 89 publications, with 30 from the Cochrane Library, 24 from PubMed, 22 from Medline, and 13 from Science Direct. Four were excluded due to insufficient emphasis on primary determinants and inaccessible full-text records. The remaining 32 papers were selected for the study, assessing their quality through a thorough examination of the abstract, title, study type, and complete text availability. Table 1 explains the excluded studies, offering a critical review of the research process, identifying gaps in the literature, and facilitating discussions on study eligibility. They are essential for transparency, assessing eligibility criteria, identifying methodological trends, sensitivity analysis, providing contextual information, and resolving conflicts. Factors leading to their exclusion include studies not focusing on primary determinants [11] and studies for which full text is not available [12-14].

**Table 1:** Excluded studies in systematic review: criteria and justifications

Author/s	Topic	Reasons for exclusion
Amao et al. (2022) [11]	Current situation and improved monitoring of monkeypox in Nigeria during the COVID-19 epidemic	Did not focus on the primary determinant
McCarthy (2022) [12]	Therapeutic strategies to address monkeypox	Full text not available
Gevirtz (2023) [13]	Pain management of the patient with the monkeypox virus	Full text not available
Ghaseminia (2023) [14]	Preventing monkeypox outbreaks: focus on diagnosis, care, treatment, and vaccination	Full text not available

Table 2 presents a summary of the included studies, elucidating the findings of this review. Researchers found that quarantine and public education worked together to drastically lower the viral peak and wipe it out in humans. It implies that a condition free of monkeypox may be reached by strictly adhering to isolation and quarantine protocols and by taking prophylactic steps to reduce the rates of human-rodent interaction. Using Pontryagin’s maximum principle, implementing preventative techniques for rodent-to-human transmission is the most cost-effective and efficient way to manage monkeypox [15-18].

**Table 2:** Descriptive analysis of included studies in the systematic review AI, artificial intelligence

Author/s	Outcome measures	Findings
Onitilo et al. (2024) [15]	Public awareness and quarantine	The study found that combining public awareness and quarantine significantly reduces the viral peak, preventing its persistence in the human population and indicating the need for greater integration.
Peter et al. (2023) [16]	Quarantine and isolation	The study suggests that a monkeypox-free state can be achieved through careful follow-up of quarantine and isolation guidelines, as well as preventative measures minimizing contact rates between humans and rodents.
Peter et al. (2023) [17]	Preventive strategies	Using Pontryagin’s maximum principle, the research found that the most efficient and cost-effective way to reduce monkeypox is to deploy preventative techniques for rodent-to-human spread.
Idisi et al. (2023) [18]	Public awareness	The model suggests an effective public awareness campaign program to reduce monkeypox disease incidence, emphasizing the need for consistency and continuous awareness measures.
Petersen et al. (2019) [19]	Vaccination	The ecological gap caused by the increasing number of humans lacking immunity to poxvirus post-smallpox vaccination has led to a susceptible population prone to secondary epidemiological cycles.
Ejaz et al. (2022) [20]	Vaccination, prevention measures, and isolation strategies	The monkeypox resurgence threatens public health due to declining immunity from smallpox vaccination, requiring effective surveillance, prevention measures, and isolation strategies to strengthen health systems and respond to large-scale outbreaks.
Diatta et al. (2023) [21]	Prevention measures	To manage and prevent the propagation of the virus, it is essential to take precautions and be ready, such as staying away from sick people or animals.
Eshun et al. (2023) [22]	Vaccination	Higher vaccination coverage and effective contact tracing improve disease-free equilibrium, reducing outbreak likelihood. Promoting vaccination programs and strengthening contact tracing are crucial for effective monkeypox control.
Addai et al. (2023) [23]	Vaccination, public health education, and personal hygiene	The study suggests that the memory index, or fractional order, can control monkeypox virus transmission dynamics with proper vaccination, public health education, and personal hygiene practices, decreasing infected individuals.
Chen et al. (2022) [24]	Vaccination and quarantine	There are eight strategies: public awareness and education, laboratory management, risk-based vaccination, quarantine of imported goods, international collaboration, storage of vaccines, case isolation, and eradicating the epidemic.
Patel et al. (2023) [25]	Gene therapy and regenerative medicine	AI is revolutionizing precision medicine by integrating multiomics data and deep learning strategies. It aids in disease detection, screening, diagnosis, and classification and may aid gene therapy and regenerative medicine, reducing health risks. AI has the potential to effectively combat monkeypox infection and improve patient clinical management in the future.
Saijo (2023) [26]	Vaccination	Third-generation smallpox vaccines, LC16m8 and modified vaccinia Ankara, protect vaccinees from human monkeypox. Immediate vaccination and mass vaccination programs in endemic areas can reduce outbreak severity and protect patients.
Najib et al. (2023) [27]	Public health education	The study indicates the necessity for a consistent and continuous awareness program and adherence to public health measures to enhance public understanding of monkeypox disease.
Ajibola et al. (2018) [28]	Personal hygiene	Hand dryers are effective for hand sanitization, but alternating current-powered ones are inaccessible in sub-Saharan African countries. A solar-powered hand dryer is crucial for preventing diseases. The design includes solar power and hand dryer components, tested for performance.
Alavian et al. (2023) [29]	Vaccination	Prioritizing resources for prevention initiatives, including the development of appropriate vaccines, is crucial to ensure the effectiveness of smallpox vaccination against monkeypox.
Alabbas et al. (2023) [30]	Vaccination, education, and corrective measures	The paper highlights the effectiveness of public health interventions, including vaccination, education, and corrective measures, in reducing monkeypox vulnerability.
Nalca et al. (2005) [31]	Preventive measures	The monkeypox virus poses bioterrorism risks, necessitating effective prevention through limiting contact with infected patients and animals as well as respiratory exposure.
Reynolds and Damon (2012) [32]	Vaccination	Smallpox has been eradicated and vaccination has ceased, leading to a surge in human monkeypox in the Democratic Republic of the Congo. The resurgence is primarily focused on the Democratic Republic of the Congo, with factors like virulence differences, communicability, and immune evasion influencing the spread of monkeypox variants. Vaccination may provide a short-term solution, but long-term solutions are needed.
Talalwah and Aldorazi (2022) [33]	Vaccination	The study reveals a 16.3% effectiveness of the smallpox vaccine in preventing monkeypox, highlighting the need for preventive measures and recommending the development of a monkeypox vaccine.
Hung et al. (2022) [34]	Vaccination	Jynneos® and ACAM2000®, two vaccines against monkeypox, are undergoing quick development. However, stopping the replication of the virus requires quick diagnosis and quarantine of sick people.
Ullah and Kabir (2024) [35]	Preventive measures and quarantine	Strict quarantine measures and protection of humans and animals significantly mitigate disease outbreaks, preventing peak occurrences and preventing animal reservoirs.
Ara et al. (2022) [36]	Vaccination	The smallpox vaccine is the only effective monkeypox immunization option, with third-generation options recommended for healthcare professionals, pregnant women, immunocompromised patients, and the JYNNEOS/IMVAMUNE vaccine.
León-Figueroa et al. (2022) [37]	Preventive measures	Monkeypox, a rapidly spreading zoonotic disease, is transmitted through sexual contact, particularly among men. Early identification and treatment are crucial for effective health policies.
Chadaga et al. (2023) [38]	Vaccination	The monkeypox outbreak has accelerated the use of AI in health science research, enhancing diagnostics, prediction, vaccine development, and drug discovery.
Gujjar et al. (2023) [39]	Vaccination and medications	New medications and vaccinations are being used for monkeypox treatment and prevention, including JYNEOS and ACAM2000 vaccines, antiviral drugs like tecovirimat, and clinical trials for vaccines like NIOCH-14.
Kandeel et al. (2023) [40]	Vaccination	ACAM2000 and JYNNEOS vaccines effectively prevent monkeypox, despite variations in action modes and adverse effects due to JYNNEOS’ nonreplicating nature.
Natami et al. (2024) [41]	Vaccination	RNA-based vaccines have the potential to replace whole-virus vaccines due to their tremendous effectiveness, safe administration, low-cost manufacture, and quick development. These vaccines use messenger RNA to create target proteins.
Shamim et al. (2023) [42]	Vaccination and medications	Cidofovir is the most studied drug for monkeypox virus infection, while tecovirimat is the most frequently used option. Brincidofovir is not preferred due to tecovirimat’s availability. Trifluridine is used for ocular manifestations. Various drugs and vaccines are under development.
Mohanty and Mohanty (2023) [43]	Vaccination	JYNNEOS™, a weakened live vaccinia vaccine, has been approved against monkeypox, and post-exposure prophylaxis is advised within four days, with preexposure prophylaxis for high-risk personnel.
Eslami et al. (2024) [44]	Vaccination	A systematic review of recent studies on monkeypox virus highlights the potential of modified vaccinia Ankara - Bavarian Nordic, with tecovirimat emerging as a prominent antiviral, despite efficacy concerns.
Endo et al. (2022) [45]	Vaccination	The study suggests that depletion of susceptibles, vaccination, and increased awareness may have contributed to smaller outbreak sizes than projected by the branching process model and that future empirical evidence and key epidemiological parameters will inform future projections.
Geevarghese (2024) [46]	Antiviral therapies and vaccinations	The monkeypox virus requires urgent development of antiviral therapies and vaccinations, despite the therapeutic benefits of prescribed pharmaceuticals.

The ecological gap caused by the increasing number of humans lacking immunity to poxvirus post-smallpox vaccination has led to a susceptible population prone to secondary epidemiological cycles. The resurgence of monkeypox threatens public health due to declining immunity from smallpox vaccination, requiring effective surveillance, prevention measures, and isolation strategies to strengthen health systems and respond to large-scale outbreaks [19,20].

The key to managing and stopping the spread of a viral infection is being prepared and taking precautions, such as staying away from affected animals or people. Higher vaccination coverage and effective contact tracing improve disease-free equilibrium, reducing outbreak likelihood. Promoting vaccination programs and strengthening contact tracing are crucial for effective monkeypox control [21,22].

Vaccination, public health education, and personal cleanliness habits may regulate the dynamics of monkeypox viral transmission, as can the memory index or fractional order. Case isolation, education and publicity, vaccine storage, risk-based immunization, import quarantine, international cooperation, laboratory management, and a total of eight tactics are planned to end the epidemic [23,24].

Artificial intelligence (AI) is revolutionizing precision medicine by integrating multiomics data and deep learning strategies, aiding in disease detection, screening, diagnosis, and classification, and may aid gene therapy and regenerative medicine, reducing health risks. Third-generation smallpox vaccines, LC16m8 and modified vaccinia Ankara, protect vaccinees from human monkeypox. Immediate vaccination and mass vaccination programs in endemic areas can reduce outbreak severity and protect patients [25-28].

Prioritizing resources for prevention initiatives, including the development of appropriate vaccines, is crucial to ensure the effectiveness of smallpox vaccination against monkeypox. The study highlights the effectiveness of public health interventions, including vaccination, education, and corrective measures, in reducing monkeypox vulnerability [29-37].

Monkeypox is transmitted through sexual contact, particularly among men, and early identification and treatment are crucial for effective health policies. The monkeypox outbreak has accelerated the use of AI in health science research, enhancing diagnostics, prediction, vaccine development, and drug discovery. New medications and vaccinations, including JYNEOS and ACAM2000 vaccines, antiviral drugs like tecovirimat, and clinical trials for vaccines like NIOCH-14, are being used for monkeypox treatment and prevention [38-46].

NOS

The NOS is a quality assessment tool that uses a star system to score studies on a scale of 0-9. The scale has eight items across three domains, and each item is graded one point, except for comparability, which can score up to two points. If a study gets a score between 7 and 9, it is of good quality. If it gets a score between 4 and 6, it is at high risk, and if it gets a score between 0 and 3, it is at very high risk of bias [47]. Table 3 explains the quality assessment of observational studies.

**Table 3:** Quality assessment of observational studies using the NOS in the systematic review NOS, Newcastle-Ottawa Scale

Study	7–9	4–6	0–3		Overall quality
	High quality	High risk	Very high risk of bias		
	Selection	Comparability	Outcome	Total score	
Onitilo et al. (2024) [15]	4	0	3	7	High quality
Peter et al. (2023) [16]	4	0	3	7	High quality
Peter et al. (2023) [17]	4	0	3	7	High quality
Idisi et al. (2023) [18]	4	0	3	7	High quality
Petersen et al. (2019) [19]	4	0	3	7	High quality
Ejaz et al. (2022) [20]	4	0	3	7	High quality
Diatta et al. (2023) [21]	4	0	3	7	High quality
Eshun et al. (2023) [22]	4	0	3	8	High quality
Addai et al. (2023) [23]	4	0	3	8	High quality
Chen et al. (2022) [24]	4	0	3	8	High quality
Patel et al. (2023) [25]	4	0	3	7	High quality
Saijo (2023) [26]	4	0	3	7	High quality
Najib et al. (2023) [27]	4	0	3	7	High quality
Ajibola et al. (2018) [28]	4	0	3	7	High quality
Alavian et al. (2023) [29]	4	1	3	8	High quality
Alabbas et al. (2023) [30]	4	0	3	7	High quality
Nalca et al. (2005) [31]	4	0	3	7	High quality
Reynolds and Damon (2012) [32]	4	0	3	7	High quality
Talalwah and Aldorazi (2022) [33]	4	0	3	7	High quality
Hung et al. (2022) [34]	4	0	3	7	High quality
Ullah and Kabir (2024) [35]	4	0	3	7	High quality
Ara et al. (2022) [36]	4	0	3	7	High quality
León-Figueroa et al. (2022) [37]	4	0	3	7	High quality
Chadaga et al. (2023) [38]	4	0	3	7	High quality
Gujjar et al. (2023) [39]	4	0	3	7	High quality
Kandeel et al. (2023) [40]	4	0	3	7	High quality
Natami et al. (2024) [41]	4	0	3	7	High quality
Shamim et al. (2023) [42]	4	0	3	7	High quality
Mohanty and Mohanty (2023) [43]	4	0	3	7	High quality
Eslami et al. (2024) [44]	4	0	3	7	High quality
Endo et al. (2022) [45]	4	0	3	7	High quality
Geevarghese (2024) [46]	4	0	3	7	High quality

Discussion

The study suggests that combining public awareness and quarantine can significantly reduce the viral peak and prevent its persistence in the human population. A monkeypox-free state can be achieved through careful follow-up of quarantine and isolation guidelines, as well as preventative measures minimizing contact rates between humans and rodents. Implementing preventive strategies for transmission is the most economical and effective method for controlling monkeypox, using Pontryagin’s maximal principle. The ecological gap caused by the increasing number of humans lacking immunity to poxvirus post-smallpox vaccination has led to a susceptible population prone to secondary epidemiological cycles.

Monkeypox, a zoonotic disease, is resurging in Nigeria due to increasing contact between humans and wildlife, particularly in northeastern Nigeria. Improving surveillance systems is crucial for effective detection and response to outbreaks. Public awareness about the disease and prevention through campaigns, community engagement, and outreach programs is also critical [48].

Factors such as rural-urban migration, international travel, pastoral farming, high treatment costs, and stigmatization of monkeypox patients in Africa hinder the eradication of the disease, highlighting the need for effective public health management measures [49].

Poor disease surveillance systems and medical laboratories in African countries, particularly the West and Central regions, have contributed to the increased impedance against monkeypox eradication. The fragile healthcare system in the African region has been weakened due to excessive strain, inadequate monitoring systems, isolation facilities, and insufficient disease testing. The unavailability of vaccines, particularly in rural areas, has further reduced efforts to eradicate monkeypox. The origin of infection is also a significant factor, with the potential for more endemic regions due to animal reservoirs that may transmit back to humans. Poor documentation of local transmission among individuals is another challenge, potentially leading to a rise in asymptomatic individuals without clinical symptoms [50].

Nigeria needs to get smallpox and monkeypox vaccinations quickly since no African country has begun immunizing against the virus. According to the research, about one million immunizations went bad because no one got the shot. The public has to be informed about vaccinations accurately to overcome vaccine hesitation. The prompt testing and identification of patients depend on sufficient diagnostic facilities. To respond effectively, healthcare providers must undergo capacity development and training. It is also crucial that healthcare providers and the general population have more information about monkeypox.

Implications of the main findings

The study’s findings on the feasibility of eliminating the monkeypox virus in Nigeria could guide targeted interventions, strengthen surveillance infrastructure, increase vaccination efforts, promote community engagement and education, strengthen healthcare systems, and encourage cross-border collaboration. These findings could inform evidence-based policies and investments to reduce disease burden, strengthen healthcare systems, and achieve disease elimination. The study’s findings could also inform strategies for preventing monkeypox transmission across borders.

Limitations

Monkeypox virus elimination in Nigeria faces several limitations due to complex transmission dynamics, diagnostic challenges, limited vaccination coverage, weak healthcare infrastructure, socioeconomic factors, cross-border transmission, community engagement, logistical and operational challenges, and emerging variants. Addressing these issues requires concerted efforts, including surveillance, vaccination, community engagement, and strengthening healthcare infrastructure. Addressing these challenges and addressing socioeconomic factors, cross-border transmission, and emerging variants can help eliminate monkeypox transmission in Nigeria.

Recommendations and future prospective

The monkeypox virus in Nigeria faces challenges in eliminating it. Recommendations include enhanced surveillance systems, community engagement and education, a strategic vaccination plan, adopting a One Health approach, cross-border collaboration, research and innovation, policy support and resource allocation, and robust monitoring and evaluation mechanisms. These measures will help Nigeria work toward eliminating the virus and safeguarding public health and well-being.

## Conclusions

The monkeypox virus reemerges in Nigeria, increasing mortality rates. Awareness programs should be conducted on its danger, transmission mode, and potential for human transmission. The study examines the epidemiology of the monkeypox virus, revealing that it became more contagious in 2022 due to a special transmission network outside Africa and potential adaptive mutations. It explores potential susceptibility to cattle, goats, sheep, and pigs and suggests that infection could be latent in some primates. The study also highlights the importance of strengthened control measures and eight strategies for elimination.
